# Effects of midsole cushioning stiffness on Achilles tendon stretch during running

**DOI:** 10.1038/s41598-022-07719-x

**Published:** 2022-03-09

**Authors:** Michael Esposito, John W. Wannop, Darren J. Stefanyshyn

**Affiliations:** 1 Human Performance Laboratory, Faculty of Kinesiology, University of Calgary, Calgary, AB Canada; 2 Biomedical Engineering Graduate Program, University of Calgary, Calgary, AB Canada

**Keywords:** Biomedical engineering, Musculoskeletal system, Tendons

## Abstract

Footwear midsole material can have a direct influence on running performance. However, the exact mechanism of improved performance remains unknown. The purpose of this study was to determine if Achilles tendon energetics could potentially play a role in the performance improvements, by testing if changes in footwear midsole stiffness elicit changes in Achilles tendon stretch. Fourteen runners ran in two footwear conditions while kinematic, kinetic, metabolic and ultrasound data were recorded. There was a moderate positive correlation between the difference in stretch and the difference in performance, which was statistically significant (r(12) = 0.563, *p* = 0.036). Twelve participants had greater stretch and better performance in the same footwear condition. Based on stretch estimates, the difference between conditions in energy returned from the Achilles tendon was 3.9% of the mechanical energy required per step. Energy return of this magnitude would be relevant and could cause the improved performance observed. These results suggest that increasing energy returned from the Achilles could be a valid mechanism for improving running performance due to changes in footwear. These findings lead the way for future research to further understand internal mechanisms behind improved running performance.

## Introduction

Running footwear research has shown that midsole material properties can have a direct influence on running performance^1–5^. Footwear midsoles have been a long-studied topic, in the mid 1980s, new cushioning materials, such as ethylene vinyl acetate (EVA), and unique cushioning systems, such as the Nike Air™ were developed specifically for running shoes to influence performance. Frederick et al.^2^ showed an airbag system reduced the oxygen cost of running by 2.4% compared to traditional foam materials. The airbag shoes had a higher deformation of the midsole and slightly higher energy return, as compared to traditional EVA foam, with the performance improvements thought to be associated with the increased energy return. Several studies have shown that a midsole with higher energy return can positively impact performance compared to traditional foam midsoles^5,14,15^.

Although there are multiple studies showing increased energy returned from the midsole can improve running economy, additional research has shown the influence of cushioning on performance to be athlete specific^17–19^ with groups of subjects performing their best in different shoe conditions. For example, Nigg et al.^17^ found no group differences between shoe conditions, viscous or elastic heel, but about a quarter of the subjects had better performance with the viscous heel, and about a quarter had better performance with the elastic heel. Even though the viscous shoe had a greater amount of energy lost in the midsole, individuals still performed better in this condition despite greater energy loss from the midsole. Further, Sinclair and Dillon^15^ found that running in a leaf spring structured midsole, specifically designed to increase the energy returned from the midsole^15^, decreased running economy by 0.5% on average compared to a traditional running shoe. These results highlight that the energy returned from the midsole may not be fully responsible for improvements in running economy.

Other theories have been used in attempt to explain the influence that midsole material properties have on running economy. A common theory is the *cost of cushioning* hypothesis^1^, stating that external cushioning may reduce the need of muscles for cushioning, resulting in reduced metabolic cost. Tung et al.^13^ provided clear support of this hypothesis, showing a reduced metabolic cost of approximately 1.5% when running on a cushioned surface (10 mm) compared to a rigid surface. Although studies have used the cost of cushioning hypothesis to explain their results, other research has found results contrary to the hypothesis^28^. Further, as previously mentioned, research has shown that changes in midsole properties have athlete-specific influences on running economy^17–19^. This suggests the cost of cushioning hypothesis may not fully explain performance improvements observed, suggesting there may be other internal mechanisms responsible for these improvements. However, there is no conclusive evidence of an internal mechanism to explain these performance improvements to date.

Even though the internal mechanism resulting in the observed performance improvements is unknown, it is speculated that changes in footwear cushioning could influence the functioning of the Achilles tendon. When running, the Achilles tendon completes a stretch–shortening cycle during each step, where elastic strain energy is stored within the muscle–tendon unit, and is used to support and propel the body forward^20–23^. The storage and release of this elastic energy is thought to be important in maintaining a low energy cost of running^23^, by reducing the work required from the muscles^24^. Moreover, research has shown that individuals with a greater energy storage capacity of the Achilles tendon (calculated as the integral of the tendon force over the tendon strain) had better running performance (lower oxygen consumption) compared to others with a lower energy storage capacity^25^. With the Achilles tendon having a significant role during running, it is reasonable to believe that Achilles tendon mechanics may be influenced by footwear cushioning and may be part of the mechanism responsible for the performance improvements previously observed. Specifically, with footwear cushioning, it is speculated that when running with compliant cushioning, a lower heel position would occur, due to increased deformation of the midsole during stance. Due to this lower heel position, a greater extension of the Achilles tendon would occur. As a result, there would be greater energy storage and return from the Achilles tendon, which would result in a decreased work demand from the main plantarflexor muscles—gastrocnemius and soleus. Since the plantarflexor muscles are the largest contributors to mechanical work generated during running^26,27^, it is reasonable to believe that the overall result could be an improved running performance.

The purpose of this study was to determine if changes in footwear midsole stiffness elicit changes in Achilles tendon stretch, in an attempt to determine a mechanism behind running economy improvements previously observed with these differences in cushioning properties. It was hypothesized that the footwear condition with better running economy for an individual will have greater Achilles tendon stretch, suggesting greater energy storage and return of the Achilles tendon, during running.

## Results

**Figure 1:**
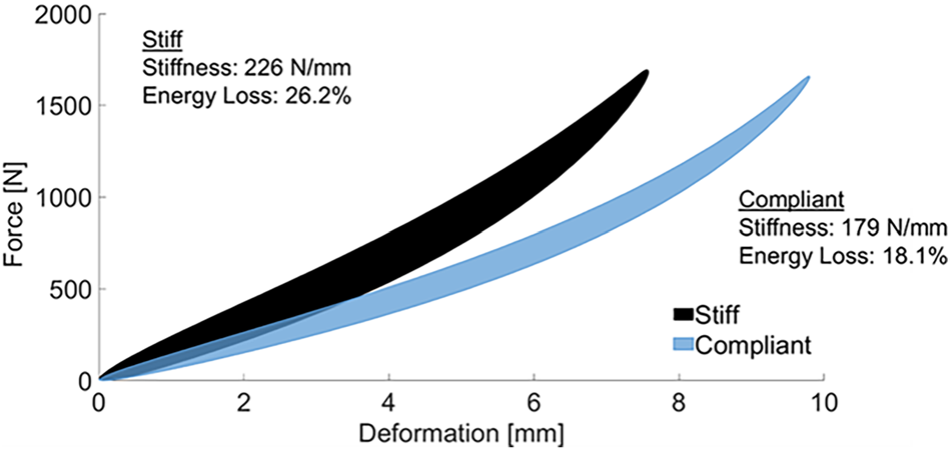
Force–deformation curves of each of the footwear conditions.

**Figure 2:**
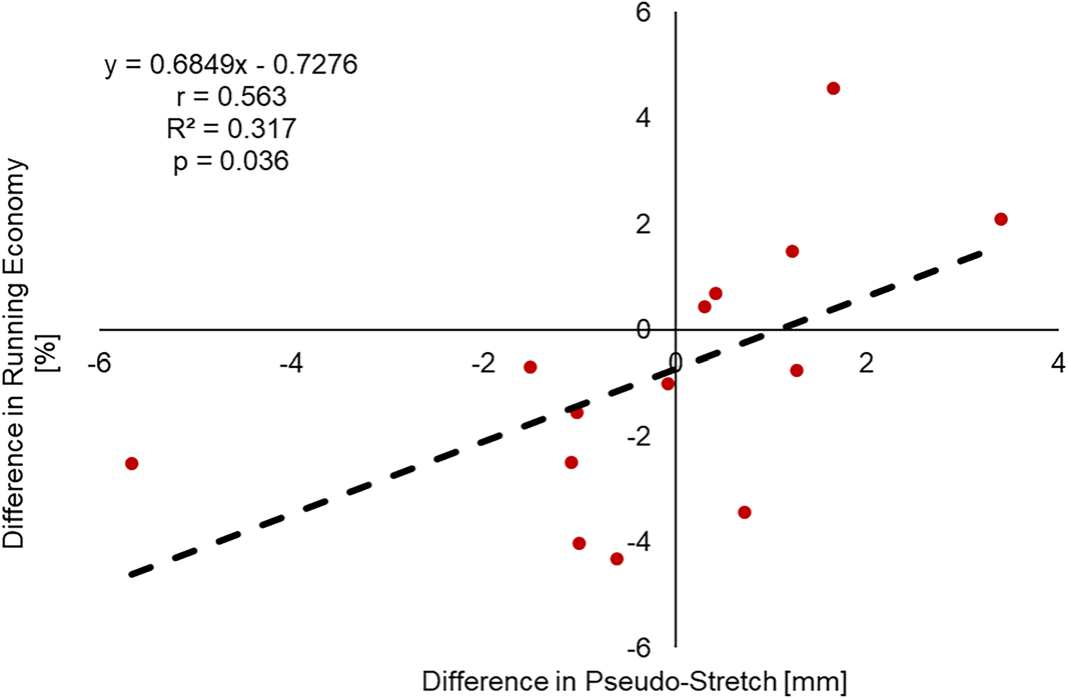
Difference in performance versus difference in pseudo-stretch (red) and trendline for all fourteen participants (black). Positive values for difference in pseudo-stretch and difference in performance indicate a greater stretch and better performance respectively in the stiff shoe condition.

From the footwear mechanical testing, the midsole of the stiff shoe was stiffer and had greater energy loss (Fig. 1). A Shapiro–Wilk test confirmed that the data were normally distributed (PS_D_: D(14) = 0.966, *p* = 0.816. ΔEC_r_: D(14) = 0.899, *p* = 0.110), therefore, parametric tests were performed. Participant values with group means and standard deviations for the variables are shown in Table 1. Positive values for difference in pseudo-stretch and difference in performance indicate a greater stretch and better performance, respectively, in the stiff shoe condition, while negative values for difference in pseudo-stretch and difference in performance indicate a greater stretch and better performance, respectively, in the compliant shoe condition. There was no group effect on pseudo-stretch (t(13) = − 0.259, *p* = 0.800) or performance (t(13) = 1.287, *p* = 0.221). There was a moderate^29^ positive correlation between the difference in pseudo-stretch and the difference in performance, which was statistically significant (r(12) = 0.563, *p* = 0.036) (Fig. 2). Twelve participants had greater pseudo-stretch and better performance in the same footwear condition and two participants (P2, P12) did not have greater pseudo-stretch and better performance in the same footwear condition. A post-hoc power analysis revealed the observed power of this study was 0.65.

**Table 1:** Participant values, group mean and standard deviations for pseudo stretch (PS) in each condition, difference in pseudo-stretch (PS_D_), energy cost of running (EC_r_) and difference in performance (ΔEC_r_).

	PS [mm]		PS_D_	EC_r_ [kJ∙kg^−1^ km^−1^]		ΔEC_r_
	Stiff	Compliant	[mm]	Stiff	Compliant	[%]
P1	24.3	30.0	− 5.7	4.59	4.47	− 2.52
P2	50.2	48.9	1.3	4.75	4.72	− 0.76
P3	44.5	45.2	− 0.6	5.09	4.87	− 4.33
P4	23.6	24.7	− 1.1	4.79	4.67	− 2.51
P5	35.9	37.4	− 1.5	4.40	4.37	− 0.70
P6	28.3	28.3	− 0.1	5.04	4.98	− 1.02
P7	40.3	38.6	1.6	4.39	4.60	4.57
P8	27.6	24.3	3.4	4.64	4.74	2.10
P9	38.2	39.3	− 1.0	4.37	4.30	− 1.55
P10	38.4	39.4	− 1.0	4.40	4.22	− 4.03
P11	38.8	37.5	1.2	3.94	4.00	1.49
P12	27	26.3	0.7	4.93	4.76	− 3.44
P13	44.1	43.8	0.3	4.71	4.73	0.44
P14	22.6	22.2	0.4	4.41	4.44	0.70
Mean	34.6	34.7	− 0.1	4.60	4.56	− 0.83
SD	8.9	8.6	2.1	0.31	0.27	2.52

## Discussion

The purpose of this study was to determine if changes in footwear cushioning stiffness can elicit changes in Achilles tendon stretch, in an attempt to determine a mechanism behind running economy improvements previously observed with differences in cushioning properties. It was hypothesized that the footwear condition with better running performance (lower oxygen consumption) would have greater energy storage and return of the Achilles tendon, attributed to a greater Achilles tendon stretch during running. In partial support of the hypothesis, there was a moderate positive correlation between the difference in pseudo-stretch of the Achilles tendon and the difference in performance between footwear conditions. This study was one of the first to determine a difference in Achilles tendon stretch due to footwear modifications and how they relate to performance, providing valuable information for future studies.

The performance results were consistent with previous studies^17–19^, with groups of subjects performing their best in different shoe conditions. In the present study, nine participants had their best performance in the compliant footwear condition. These results suggest that the cost of cushioning hypothesis may not fully explain the performance results observed, since this hypothesis would suggest that all participants would have their best performance in the compliant footwear condition due to the mechanical properties of the midsole. This is not to say that cushioning was not involved, but this theory may not fully explain performance improvements observed, suggesting there may be other internal mechanisms responsible for these improvements. These results provide some initial evidence that Achilles tendon stretch may be an internal mechanism partially responsible for the performance improvements observed.

The results show a moderate positive correlation between the difference in performance and the difference in pseudo-stretch; however, from this data, the absolute stretch of the tendon cannot be determined, and the energy stored and returned from the Achilles tendon is unknown. Intuitively, the footwear condition with greater stretch would have a greater energy storage and return from the tendon. Fletcher and MacIntosh^23^ estimated the energy returned from the Achilles tendon of trained males during running. Since the participants in that study were a similar height and mass to the participants of the present study (Fletcher and MacIntosh 1.76 m, 67.8 kg; this study 1.75 m, 67.9 kg) and participants ran at a similar average speed (Fletcher and MacIntosh 3.64 m/s; this study 3.80 m/s), data from that study were used to estimate Achilles tendon energetics in this study. The energy returned from stretching the Achilles tendon was 42 J on average, ranging from 25 to 70 J^23^. Based on this estimate (42 J), assuming the hysteresis to be 10%^30^, approximately 22.3 mm of tendon stretch would occur each step. Exploring three values for increase in stretch, 0.1 mm (smallest difference in pseudo-stretch from this study, P6), 1.4 mm (absolute average of pseudo-stretch from this study) and 5.7 mm (greatest difference in pseudo-stretch from this study, P1), would result in a total tendon stretch of 22.4, 23.7, and 28.0 mm respectively. The estimated energy return from these tendon stretches are 42.4, 47.4 and 66.2 J, equating to an average difference of 5.4 J ranging from 0.4 to 24.4 J. The average mechanical energy for each running step was estimated as 140 J, using mechanical power estimates from Williams and Cavanagh^31^. Based on this, the difference in energy return from the Achilles tendon would result in an average of 3.9% of the total mechanical energy required, ranging from 0.3 to 17.3%. Energy returns of this magnitude would be relevant and could cause the improved performance observed. These results suggest that the energy returned from the Achilles could be a valid mechanism for improving performance due to changes in footwear.

The relationship between mechanical energy and metabolic energy is very complex^31^. Muscular efficiency, defined as the ratio of mechanical power to metabolic energy expenditure^31^, has been used to relate mechanical energy to metabolic energy. Using a muscular efficiency of 0.2^31^, it is estimated that a mechanical energy return of 5.4 J would correspond to 27 J of metabolic energy. Using a metabolic cost of running of 850 J/stride^23^, 27 J would equate to a 3.18% average change in metabolic energy, slightly greater than the metabolic changes of this study and previous studies examining metabolic changes due to footwear^2,5,13^. Further, it is possible that the estimations of total Achilles tendon stretch were overestimated, based on previous studies showing smaller tendon stretch^32,33^. Assuming stretch is overestimated by 50%, a 22.3 mm stretch would become 14.9 mm, returning approximately 18.8 J. Using the average increased stretch of 1.4 mm, the estimated energy return would be 22.5 J, a difference of 3.7 J and on average a total of 2.6% of the mechanical energy required. This lower percentage of mechanical energy does not change the interpretation and it is believed the energy returned from the Achilles tendon may be a valid mechanism in improving performance with changes in footwear. The results provide some initial evidence of this mechanism, but further research is needed to fully understand this mechanism.

Although the results of the present study suggest that the energy returned by the Achilles tendon may be a valid mechanism of improved running performance, the possibility of other mechanisms cannot be discounted. The stretch of the Achilles tendon may result in changes to the function of the triceps surae. If the length change of the muscle tendon unit during a stride is taken up by stretching of the Achilles tendon, this allows for less shortening of the muscle fascicles and slower shortening velocity^22,34^. Less shortening of the muscle fascicles allow for optimization of muscle activation and force–length-velocity properties^35^. It is believed that the optimization of these relationships could result in a reduced metabolic cost, however, more research is required.

There are a few limitations that should be noted. Absolute tendon stretch was not calculated. Without absolute tendon stretch, energy storage and return values of the Achilles tendon can only be estimated. In this study, the difference of tendon movement between conditions was calculated, and it is believed that this difference would equal the difference in stretch between conditions. This data set shows that changes to footwear cushioning may be able to elicit changes in Achilles tendon stretch, and further research is needed to determine the exact energy savings related to the increase in stretch. Moreover, future work should investigate the main reasons why individuals have greater tendon stretch and perform better in certain shoe conditions compared to others.

## Conclusions

This study investigated the effect of midsole cushioning on Achilles tendon stretch while running. It was speculated that increased stretch of the Achilles tendon would return more energy and result in an improved running performance. There was a positive moderate correlation between difference in performance and difference in tendon stretch between footwear conditions. The estimated difference in energy returned by the tendon was large enough to result in improvements to performance. The results of this study suggest that increasing energy returned by the Achilles tendon may be a valid mechanism to improve running performance. These findings lead the way for future research to further understand the mechanism behind improved performance. Understanding how footwear modifications affect internal mechanisms could have large ramifications on potential strategies for assisting and supporting locomotion.

## Methods

### Participants

Fourteen male participants (Age: 26.1 ± 6.1 years, Height: 1.75 ± 0.06 m, Mass: 67.9 ± 8.4 kg) with personal best 10 km run times of less than 40 min were recruited for the study. All participants fit into a US 9 shoe size and were free from lower extremity injury or pain at the time of testing. Individuals received verbal and written descriptions of the study design and informed written consent was obtained from all individuals prior to data collection. This study was approved by the University of Calgary Conjoint Health Research Ethics Board (REB18-0924). All experiments were performed according to the approved protocol and in accordance with the ethical standards of the Declaration of Helsinki.

### Footwear

Two footwear conditions were used—stiff and compliant. The stiff shoe had a midsole made of EVA and the compliant shoe’s midsole was made of expanded thermoplastic polyurethane pellets (Boost). Concerted effort was made to construct the shoes such that they were identical in every aspect except for the midsole cushioning material. With the different midsole materials, the shoes had a small difference in mass (Stiff: 311 g, Compliant: 322 g); therefore, the shoes were matched in mass by evenly distributing lead weights near the center of mass of the stiff shoe to eliminate any potential confounding effect of mass. These two shoes were chosen since previous research has used footwear with similar differences in cushioning stiffness and observed differences in running economy of 1% on average between footwear conditions^5^.

### Footwear mechanical testing

Rearfoot cushioning stiffness (compressive stiffness) and energy loss of the footwear was measured using a previously published protocol^5^. Each shoe condition was subjected to three test sessions with each session consisting of 20 consecutive loading and unloading cycles. Each cycle consisted of the shoe being compressed at a rate of 4250 N/s until a maximal load of 1700 N was reached, then the shoe was unloaded. For each shoe, average stiffness and hysteresis values were calculated using the 20th loading–unloading cycle from each of the three test sessions and averaged.

### Data collection

**Figure 3:**
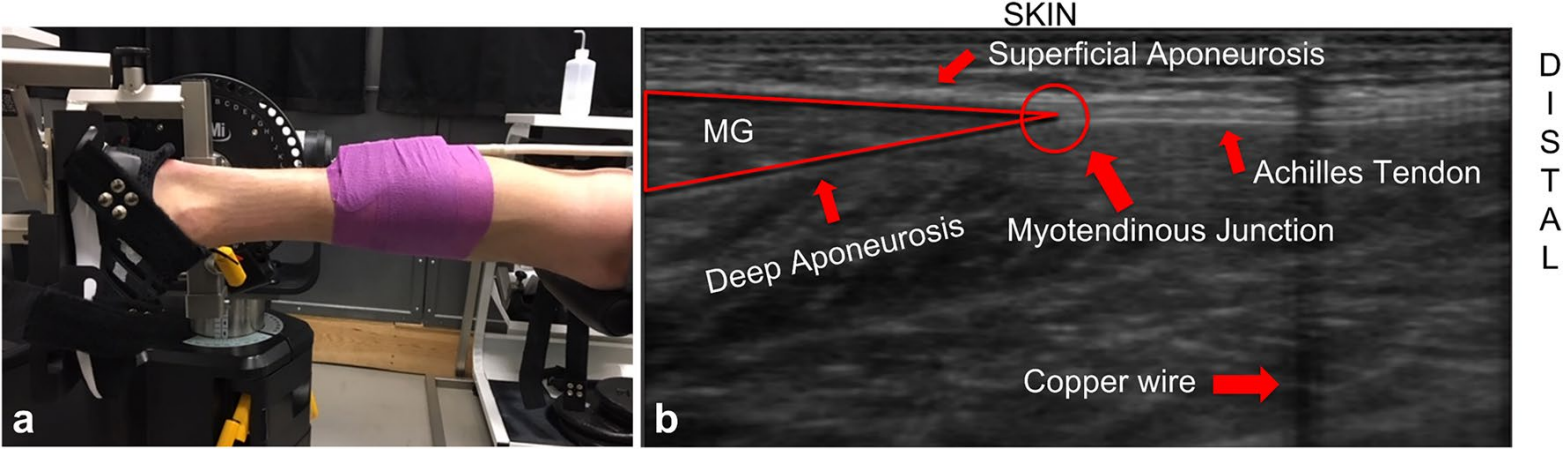
(**a**) Positioning of the participant in the Cybex with the ultrasound probe attached. (**b**) Labelled ultrasound image when probe was secured to the participant.

Testing was completed during two sessions. The objective of session one was to measure Achilles tendon moment arm, using a previously published method^36^. Participants laid prone with their knee at 180° (straight leg) and their ankle at 90° in a dynamometer (Cybex NORM, Humac, CA, USA) (Fig. 3). The axis of rotation of the Cybex was aligned with the ankle joint, while the participant’s left foot was secured using Velcro straps. Then, participants were fitted with an ultrasound probe (LV8-5N60-A2 transducer, ArtUs EXT-1H, Telemed, Lituania). The ultrasound probe was positioned to measure the movement of the myotendinous junction of the left medial gastrocnemius (Fig. 3). A copper wire was placed on the skin underneath the ultrasound probe. The wire was visible in the ultrasound field of view and was used to determine if the probe moved during the testing session. The probe was placed in a custom 3D printed holder, designed to prevent the probe from rotating off the skin, and was held in place with athletic wrap and gaffer tape. To determine the moment arm of the Achilles tendon, the ankle was passively rotated in the Cybex from 30° to 0° plantarflexion at 5°/s. Three repetitions served as conditioning trials, and on the fourth trial, ultrasound images were recorded.

Session two was a treadmill running session and was conducted on an instrumented treadmill (Bertec Corporation, Columbus, USA) set at a 1° incline to mimic outdoor running^37^. The protocol for session two was adapted from previously published methods^4^. The participants were fitted with the ultrasound probe in the same position as session one. The probe was secured by first taping the probe to the skin and then wrapped with athletic wrap. After the ultrasound probe was secured, the test session began with an incremental running test to a submaximal intensity to determine at which speed the participant would reach their anaerobic threshold. A metabolic cart (Cosmed, Rome, Italy) was used to measure breath-by-breath pulmonary gas exchanges. Participants started the test at 2.46 m/s and the speed increased 0.22 m/s every two minutes. The incremental test continued until each athlete reached their anaerobic threshold (via monitoring of the real-time Cosmed output). Anaerobic threshold was visually identified from the real-time data feed by looking for a combination of excessive CO_2_ production^38^, a value of respiratory exchange ratio greater than 1^39^ and a non-linear increase in pulmonary ventilation. The anaerobic threshold was used to determine the submaximal running speed for the testing session, and ensure the speed was relative to each participant’s fitness level. The ultrasound probe was attached during the incremental test, allowing subjects to adapt to running with the probe prior to data collection. However, no kinematic, kinetic, or ultrasound data were recorded during the incremental test. The incremental test also allowed each participant to warm up prior to data collection, and participants were given a 10-min rest following completion of the incremental test before the running trials.

**Figure 4:**
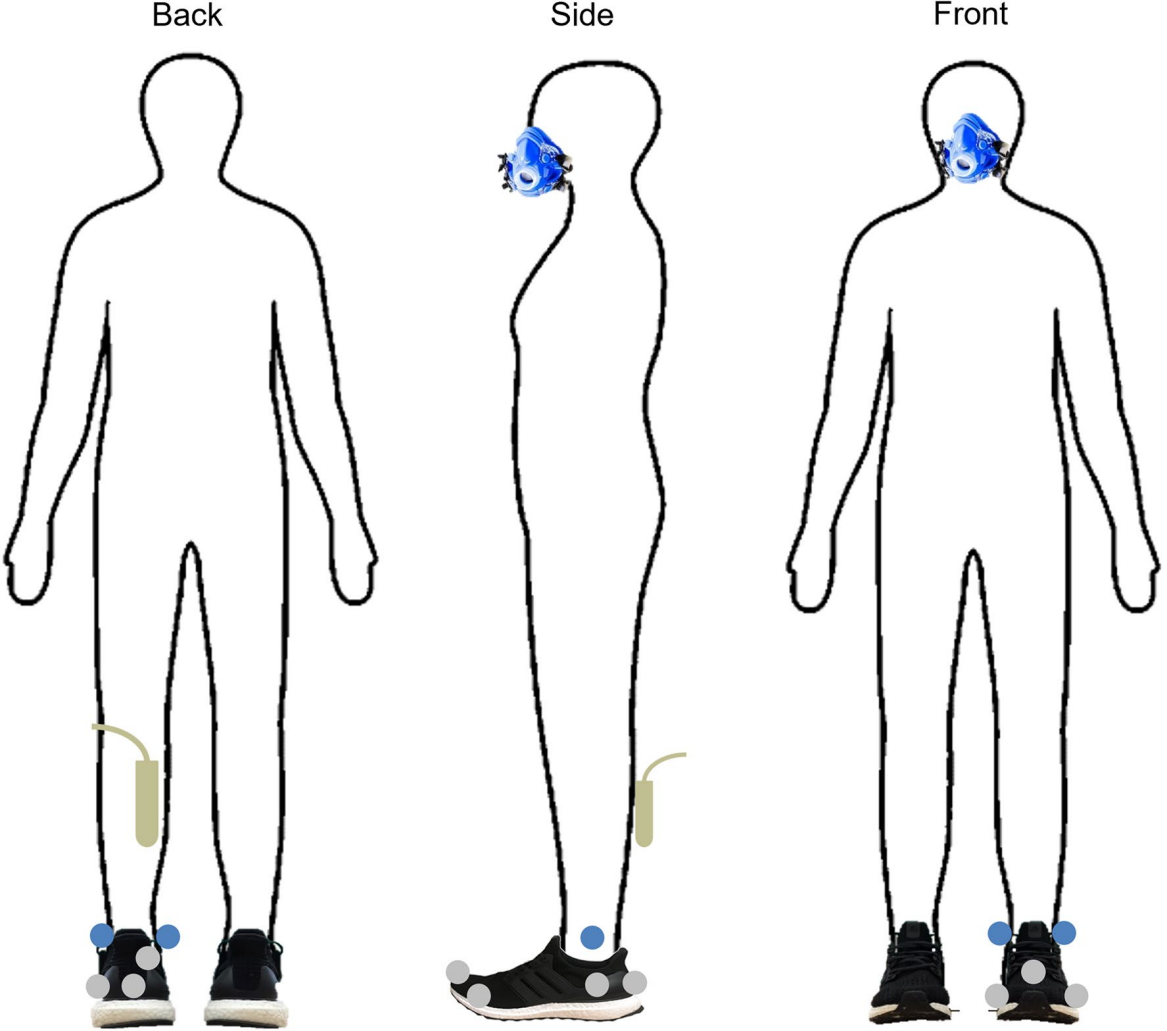
Schematic views of the back (left), side (middle) and front (right) view of the participant with all equipment attached during the running trials (motion capture markers, ultrasound probe and breathing mask). The grey markers remained on while the blue markers were removed during the running trials.

During the running trials, kinematic, kinetic, metabolic and ultrasound data were collected. Kinematic data were measured with the use of an 8-camera motion analysis system (200 Hz) (Vicon Motion Systems Ltd., Oxford, UK). Ten retroreflective markers were placed on each participant and used to track the motion of the rearfoot and forefoot through space (Fig. 4). The marker positions consisted of four anatomical landmark markers and six tracking markers. The anatomical landmark markers were placed on the left medial and lateral malleoli, left 1^st^ metatarsal and the left 5^th^ metatarsal. The tracking markers included a cluster of three markers placed on the forefoot and a cluster of three markers placed on the rearfoot aspects of the shoe. Ground reaction force data were obtained with an instrumented treadmill (1000 Hz), and metabolic data were recorded breath by breath using the metabolic cart. The ultrasound (177 Hz) was recorded with the following settings: Depth: 30 mm, Dynamic Range: 72 dB, Power: -3 dB, Frequency: 8 MHz.

Once all the markers were attached, a static motion capture trial was recorded prior to the running trials to determine joint centers; the malleoli and metatarsal markers were subsequently removed for the running trials. Participants performed two 6-min steady state running trials at a submaximal running speed (average speed = 3.80 ± 0.49 m/s), one in each shoe condition in a random order. The running speed of the trials was determined from the incremental test, 0.22 m/s slower than the final speed of the incremental test, to ensure the participant was running at a speed below their anaerobic threshold. The first four minutes of the running trial allowed the participant to reach a steady-state of oxygen consumption^40^, and data from all systems (kinematics, kinetics, metabolic and ultrasound) were recorded during the final two minutes of the trial. Participants were given a 10-min rest following completion of the first running trial before undertaking the second running trial.

### Metabolic data

The energy cost of running (EC_r_) (kJ∙kg^−1^∙km^−^1) was calculated from the equation presented by Fletcher et al.^36^ and was calculated as the average of every breath during the final 2 min of the trial. Differences in performance were calculated as the percent difference between conditions.

### Ultrasound data

**Figure 5:**
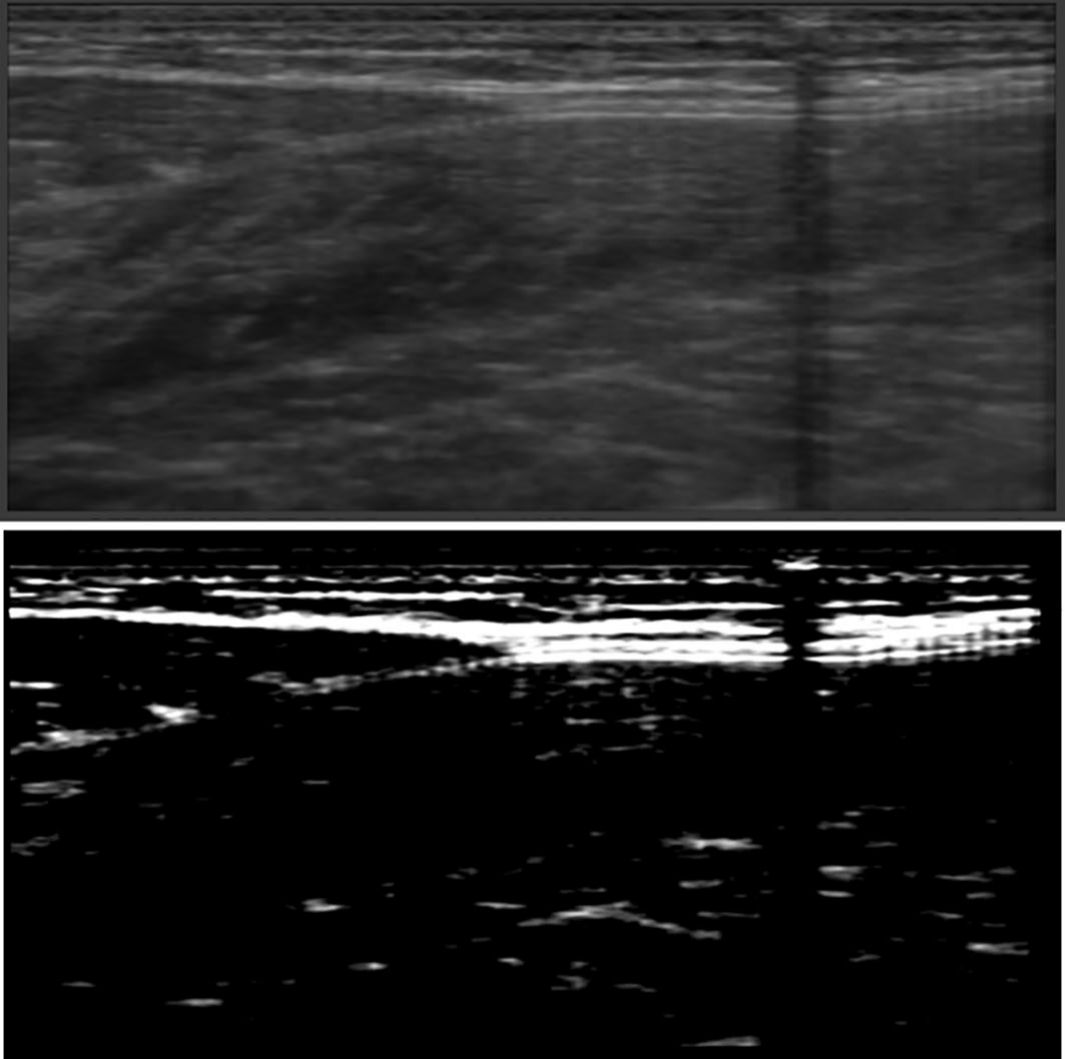
Example of ultrasound image before (top) and after (bottom) post-processing in ImageJ.

The ultrasound videos were saved as TVD files (file format specific to ultrasound software) during the data collection. The ultrasound videos were then converted to a series of images (PNG files) to be post-processed. The ultrasound images were post-processed using ImageJ (Version 1.53a, National Institutes of Health, Bethesda, MD, USA, URL: http://imageJ.nih.gov/ij). The contrast and brightness were all adjusted using ImageJ (Fig. 5). This was done to enhance the contrast between the muscle tissue and the aponeurosis of the muscle. The Achilles tendon moment arm was calculated using the tendon excursion method^41^ from when the ankle was passively rotated. Myotendinous junction displacement was quantified in ImageJ and displacement measured was converted from pixels to millimeters from the scale determined in ImageJ. The moment arm was then adjusted to account for differences in values obtained from the tendon excursion method and the center of rotation method based on data from Fath et al. (2010). There is no gold standard for moment arm measurement, but it has been shown that tendon excursion method results in smaller moment arm estimate by an average of 15 mm compared to the center of rotation method ^41^.

From the ultrasound images recorded during the running trials, movement of the myotendinous junction (M_MTJ_) was measured as the distance between the copper wire position and the most proximal myotendinous junction position. All distances were measured thirty times per condition and the distances measured were converted from pixels to millimeters.

### Kinematic data

Kinematic data were analyzed using Visual 3D software (v2020.10.03, C-Motion, Germantown, MD, USA, URL: https://c-motion.com/). Ground reaction force and kinematic data were smoothed using a zero-lag low-pass 4th order Butterworth filter with a cut-off frequency of 50 Hz. A 20-N threshold for the vertical ground reaction force was used to identify the stance phase of running, and thirty strides for the left leg were extracted for the analysis of each shoe condition. The joint center for the ankle was determined as the midpoint between the lateral and medial malleoli markers in the neutral trial. Cardan angles were calculated for the ankle joint with a flexion–extension, abduction–adduction, and internal–external rotation sequence ^42^. Movement of the Achilles tendon at the ankle (M_Ank_) was calculated from the following equation:

1 $${M}_{Ank}= {r}_{AT}\mathrm{tan} \, \theta$$

where ${r}_{AT}$ is the Achilles tendon moment arm and $\theta$ is the dorsiflexion angle relative to the ankle angle during the static trial.

### Pseudo-stretch of Achilles tendon

Pseudo-stretch of the Achilles (PS) was calculated by adding M_Ank_ and M_MTJ_ together for each footwear condition. The variable was termed “pseudo-stretch” since the true stretch of the tendon cannot be determined, and it was used to find differences between conditions. The difference between conditions of pseudo-stretch is thought to be the difference in Achilles tendon stretch, not the total amount of stretch.The difference in PS (PS_D_) was calculated as PS in the stiff condition minus PS in the compliant condition.

### Statistics

All statistical analysis was done using SPSS (v26, IBM, Armonk, NY, USA) and values of *p* less than 0.05 were considered statistically significant. The Shapiro–Wilk test was performed to confirm that the variables (PS_D_ and ΔEC_r_) in each shoe condition were normally distributed. Based on the assumption that the distributions were normal, a paired T-test was used to assess differences between footwear conditions and a Pearson correlation analysis was performed to assess the existence of a linear relationship between the difference in performance and the difference in pseudo-stretch measurements.
